# Challenging Decision-Making Between Transcatheter Aortic Valve Implantation and Aortic Valve Surgery: A Case of a Jehovah’s Witness Patient With Severe Symptomatic Aortic Stenosis Coexisting With Severe Mitral Regurgitation and Bicuspid Aortic Valve

**DOI:** 10.7759/cureus.34973

**Published:** 2023-02-14

**Authors:** Osagioduwa Mike Atoe-Imagbe, Abdulrahman Azzu, Henry O Aiwuyo, John O Osarenkhoe

**Affiliations:** 1 Medicine, Delta State University Teaching Hospital, Oghara, NGA; 2 Medicine, Betsi Cadwaladr University Health Board, Bangor, GBR; 3 Cardiology, Ysbyty Gwynedd, Bangor, GBR; 4 Internal Medicine, Brookdale University Hospital Medical Center, Brooklyn, USA; 5 Medicine and Surgery, Igbinedion University Teaching Hospital, Benin City, NGA

**Keywords:** multidisciplinary decision-making, jehovah's witness, bicuspid aortic valve disease, mitral regurgitation (mr), aortic stenosis (as), aortic valve surgery, transcatheter aortic valve implantation (tavi)

## Abstract

A 73-year-old Jehovah’s witness man with a bicuspid aortic valve and a history of epilepsy presented to the emergency room with chest pain and dyspnea. Echocardiography revealed normal left ventricular systolic function, but also revealed severe aortic stenosis and severe mitral regurgitation. Coronary angiography and computerized tomography angiography ruled out any significant coronary artery disease and aortic dissection, respectively. In view of his religious views, transcatheter aortic valve implantation was considered more suitable than aortic valve surgery and was successful with a stable postoperative state. This case reaffirms that autonomy should be maintained while considering the best interest of patients in decision-making.

## Introduction

Decision-making on the best management approach in patients with complex aortic valve disease can be challenging. The most common of this clinical scenario is a combination of severe aortic stenosis (AS) together with severe mitral regurgitation (MR) [1], which can be challenging in deciding which treatment approach to follow. This is further complicated if the patient has complex anatomies, such as a bicuspid aortic valve (BAV), a single coronary artery, or where situs inversus coexists in these patients [2,3]. The coexistence of MR with severe AS is well documented and, in several instances, this has led to difficulties in deciding which intervention is preferred [2]. Management of patients with combined AS and MR is associated with difficulties during the preoperative assessment of patients, the choice of intervention, and when considering postoperative complications, and subsequent follow-up.

Transcatheter aortic valve implantation (TAVI) is considered safe in well-selected patients with AS, according to PARTNER 1 trial [2]. However, this procedure poses some difficulties in certain patient groups. Well-defined exclusion criteria make the procedure unsuitable for many patients with comorbidities associated with AS. It was initially a modality for treatment in cases of severe symptomatic AS that were either inoperable or in high-risk surgery patients. However, recent advancements in skills and experience with the procedure have resulted in the expansion of the indications for TAVI. In all TAVI studies, the procedure was performed electively and one of the consistent exclusion criteria has been unicuspidal and BAVs due to difficult anatomy as well as the associated high risks and uncertain outcomes [4].

This report presents a successful TAVI in a 73-year-old Jehovah’s witness with multiple valve pathologies after a delay in deciding the treatment approach, and the evidence for the choice of treatment is discussed as well.

## Case presentation

**Figure 1:**
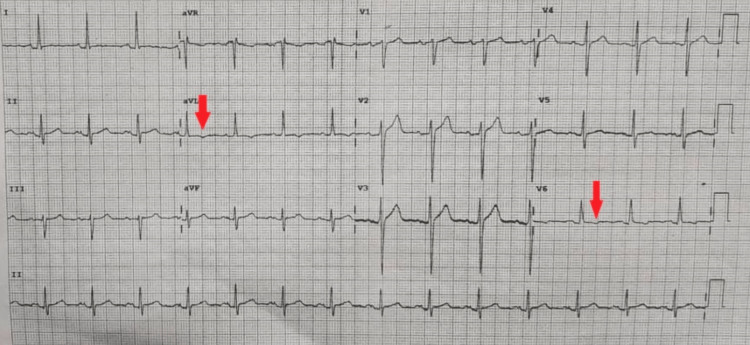
Electrocardiogram Arrow indicates T-wave inversions in the anterolateral leads

Mr. H.R. is a 73-year-old Jehovah’s witness who presented with severe recurrent chest pains over the course of two years which became intolerable 72 hours prior to presentation. The chest pain was described as tearing in nature, central in location, radiating to the back, and associated with shortness of breath which was worse on mild exertion. These symptoms in a patient with a BAV suggest symptomatic AS. He was previously diagnosed with epilepsy for which he was being followed up in the outpatient clinic. His epilepsy was previously controlled with his antiepileptic medications (sodium valproate and levetiracetam) until the day of presentation when he had a fit, an episode that was attributed to the night dose he missed while being evaluated in the emergency department. The patient lives with his wife and is well supported by his family. He has no surgical history, is not diabetic or hypertensive, and has no significant smoking or alcohol history. On examination at presentation, he was in painful distress, with no pedal edema. His heart rate was 96 beats per minute and his blood pressure was 119/83 mmHg. His jugular venous pressure was not elevated. The first and second heart sounds were present and regular, with an ejection systolic murmur, heard loudest at the aortic area, radiating to the carotids. These features were clinically suggestive of severe AS. The chest was clear, with no significant abdominal signs and no focal neurologic findings. He was subsequently admitted to the cardiology ward for evaluation. The initial electrocardiogram showed no signs of acute myocardial infarction except for T-wave inversions in the high lateral leads, with poor R-wave progression (Figure 1).

**Figure 2:**
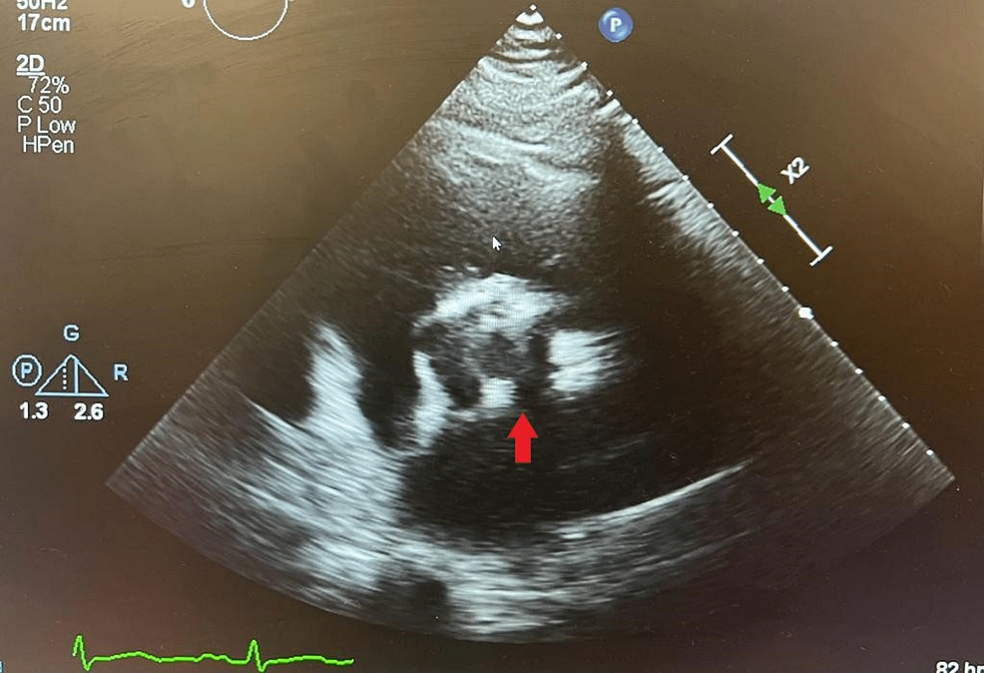
Transthoracic echocardiogram Arrow indicates heavily calcified bicuspid aortic valve

Troponin-I at presentation was 12 ng/L, and a repeat done 6 hours later was 28 ng/L. Urgent computed tomography of the aorta done at presentation showed severe AS with mild post-stenotic dilatation of the ascending thoracic aorta, without evidence of dilatation. Echocardiography showed a dilated left atrium with an unindexed biplane volume of 80 mL. It shows a normal-sized left ventricle with preserved systolic function and an ejection fraction of around 55-60%. It also showed a BAV with marked calcification and reduced cusp mobility (Figure 2), with a mean pressure gradient of 66 mmHg and a peak gradient of 109 mmHg, velocity of 5.22 m/s.

**Figure 3:**
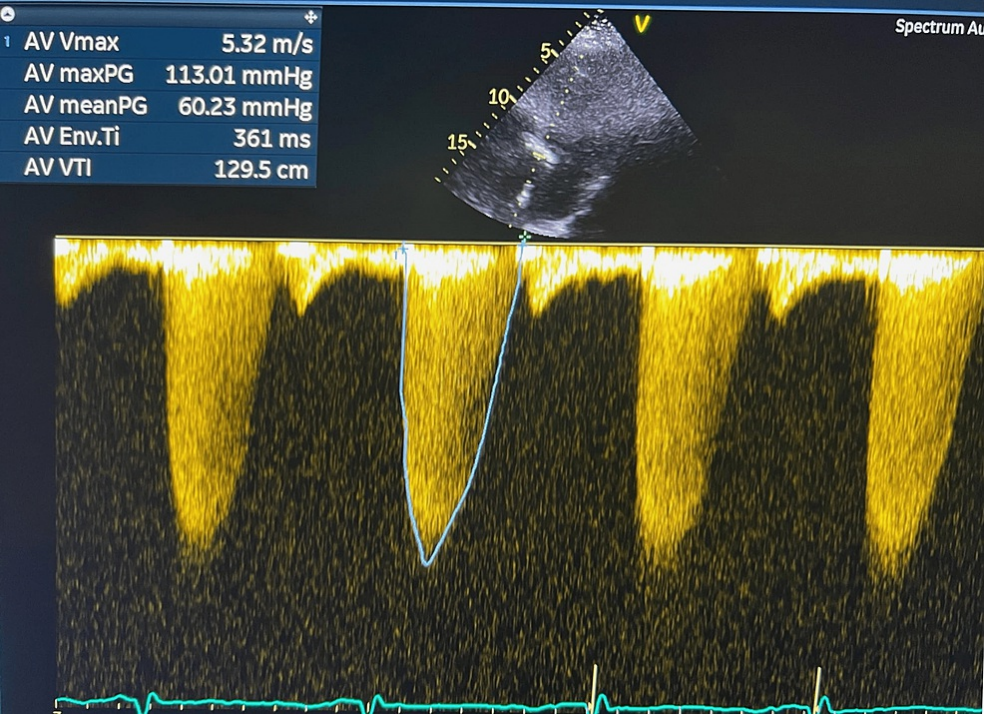
Transthoracic echocardiogram showing severe aortic stenosis with a mean aortic valve gradient of 60 mmHg and a maximum gradient of 113 mmHg

The aortic valve area (AVA) by continuity equation was 0.7 cm^2^, and there was no significant aortic regurgitation (Figure 3). 

**Figure 4:**
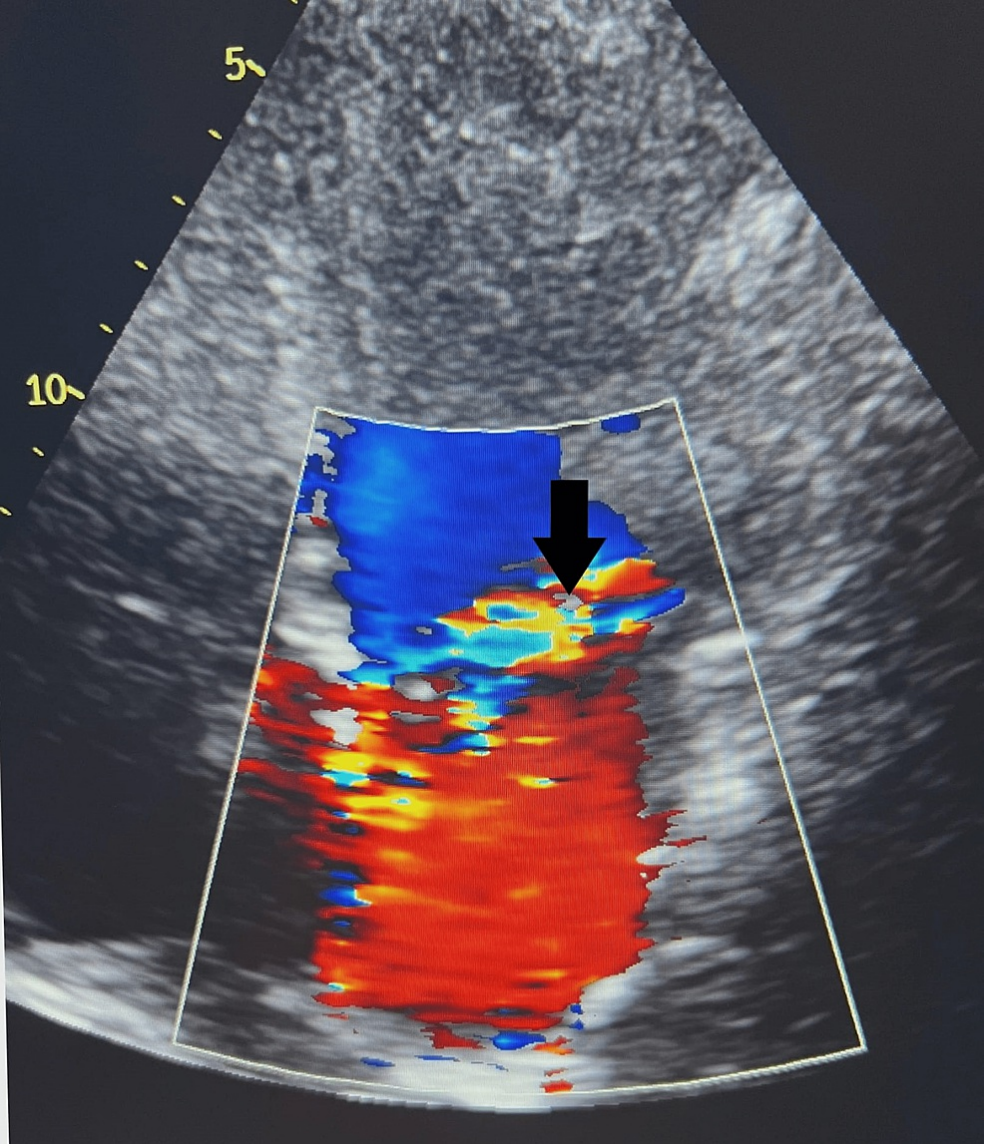
Echocardiography Arrow indicates severe mitral regurgitation

The mitral valve appeared thin and unrestricted in motion with no evidence of stenosis; an eccentric anteriorly directed MR jet was noted, with difficulty in assessing the severity of MR (Figure 4).

**Figure 5:**
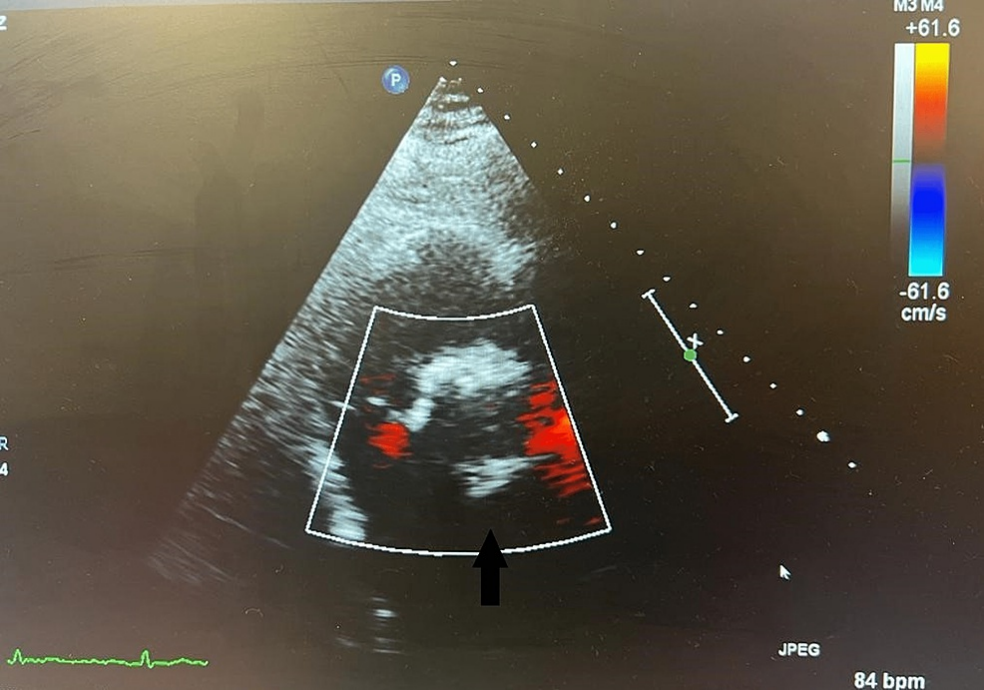
Echocardiography Arrow indicates implanted aortic valve

A coronary angiogram showed a dominant right coronary artery, which was unobstructed, with a non-dominant circumflex, and no evidence of obstructive coronary artery disease. Symptomatic AS, acute coronary syndrome, and aortic dissection were the topmost of all the considered differential diagnoses. Since the patient was previously known to have a BAV, his symptoms were considered as AS. He was initially placed on the acute coronary syndrome treatment protocol in addition to his antiepileptic medications at presentation in the emergency department after ruling out aortic dissection, due to his elevated troponins in the setting of chest pain with T-wave inversions on electrocardiogram. This was discontinued after the coronary angiogram came out negative for significant coronary artery disease. The patient was then considered for the next guideline-directed treatment which is aortic valve replacement (AVR) [5] considering the presence of clinical symptoms and echocardiographic parameters showing severe AS. Surgical AVR was considered the treatment of choice in the setting of multiple valvular abnormalities. However, it was avoided due to the possible need for blood product support in this patient who has already refused consent to transfusion of blood or blood products, if needed. TAVI was previously declined due to the presence of a BAV which is a well-defined exclusion criterion [2]. A subsequent cardiac multidisciplinary team meeting thereafter concluded that the patient would benefit from a TAVI, and this was consented to by the patient. A balloon valvuloplasty was performed by advancing a balloon via the right femoral artery sheath, across the aortic valve to clear the stenosis and to deploy the 29-mm Edward SAPIEN^TM^ bioprosthetic aortic valve, which expanded within the native aortic valve (Figure 5).

**Figure 6:**
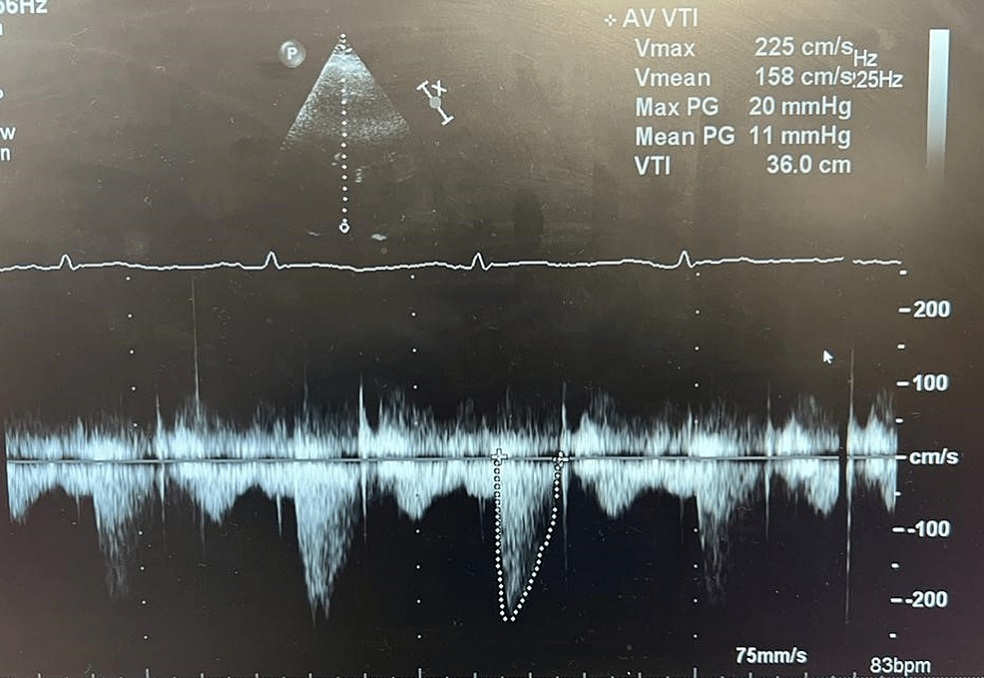
Aortic valve on echocardiography post-transcatheter aortic valve implantation, showing a mean aortic valve gradient of 11 mmHg and a maximum gradient of 20 mmHg

The mean valvular pressure gradient after TAVI decreased to 11 mmHg while the peak gradient was 20 mmHg (Figure 6). There were no intraoperative complications.

A day after TAVI, our patient’s symptoms had reduced significantly, his vital signs were stable, and a transthoracic echocardiogram (TTE) performed on the first postoperative day showed that the prosthetic aortic valve was well seated without any aortic regurgitation. A 12-lead electrocardiogram also revealed no significant changes. Since the patient did not have any indication of anticoagulation, he was placed on aspirin 75 milligrams daily to prevent thrombosis of the bioprosthetic valve. He was continued with his regular antiepileptic medications and was discharged home the next day post-procedure. One week after the TAVI, a phone conversation with the patient revealed his chest pains and exertional dyspnea had resolved. A further follow-up was scheduled for 30 days post-intervention.

## Discussion

AS is the most common valvular heart disease in the UK [6]. The prevalence of AS in men and women over 55 years of age in the United Kingdom was 1.48% in 2019 [7]. A study carried out by Frey et al. showed that over 80% of these patients present with severe symptoms, and shortness of breath is the most common symptom [8].

Calcific aortic valve disease is age-related, and its prevalence is increasing rapidly in high-income countries [9]. In these countries, BAV is more common than calcific AS among the younger population. On the other hand, the rheumatic aortic valve constitutes most cases in developing countries of the world [6].

Cases of AS frequently present in combination with other valvular pathologies, of which MR is the most common. The coexistence of these valvular pathologies makes both diagnostic and therapeutic considerations complex [1].

Assessment of MR in the presence of AS is clinically challenging. A TTE is a valuable tool in the diagnosis of valvular disease [10]. MR can lead to mitral annular dilation, leaflet tethering, or both [1]. In addition, there is increased systolic left ventricular (LV) pressure in association with AS, leading to a higher systolic trans-mitral gradient that may increase the color flow jet area, resulting in an overestimation of MR [10]. Consequently, a high color flow jet area with reduced vena contracta and/or estimated regurgitant orifice area will wrongly reflect non-significant MR [10]. A regurgitant volume and fraction can reliably assess MR severity in the setting of AS. However, degenerative changes in the mitral annulus can lead to imprecise mitral inflow orifice estimation leading to another difficulty [2,4].

Transesophageal echocardiography (TEE) diagnosis of severe AS is based on the calculation of the AVA, which may be incorrect due to operator-dependent, technically limited estimation of the outflow tract area [6]. Consequently, TTE or TEE planimetry of the aortic valve is of limited value if MR is severe and forward driving pressure is reduced [5].

Association of MR with aortic regurgitation has significant morbidity and mortality implications [1,11]. Some TAVI patients who remain symptomatic from MR can undergo staged percutaneous procedures to treat MR, especially in patients with low-gradient severe AS and moderate-to-severe primary MR, who tend to have higher mortality after TAVI [11]. A review of the literature showed that several studies consistently reported that coexisting severe MR affects both immediate and long-term mortality outcomes [1,11,12]. MR regression after successful TAVI has been documented [12,13]. This is believed to be a result of decreased trans-mitral systolic gradient, LV remodeling, and regression of LV hypertrophy as well as end-diastolic volume, causing reduced tethering forces on the mitral leaflets [11,12]. Sannino et al. reported significant improvement in MR parameters after TAVI in patients who had a successful procedure [12]. Improvement in MR of at least one grade is reported in 50-60% of patients after TAVI [13]. It is, therefore, necessary to follow up with our patient who incidentally also refused any mitral intervention at this time, for a possible regression of his MR.

Patients with AS may also present with other coexisting anatomic pathologies that can influence treatment options. Examples of such associated anatomical defects include single coronary artery, BAV, situs inversus, and anomalous coronaries [3]. Sometimes, it is also difficult to differentiate chest pain in worsening AS from acute coronary syndrome [6]. This is partly because they may present in a similar way or that both pathologies can coexist in the same patient. Although there was a mild increase in serum troponins, the absence of smoking history, hypertension, diabetes, and overweight in our patient was also not suggestive of an acute coronary syndrome. In addition, the examination findings, imaging results, and the absence of new electrocardiographic ST-segment changes, together with the negative finding on coronary angiogram were not in keeping with an acute coronary syndrome as the main reason for his symptoms. At presentation, the patient described a tearing pain radiating to the back, leading to the suspicion of aortic dissection occurring in the setting of the BAV. Aortic dissection is a known complication of a BAV [5]. Hence, an urgent computerized tomography scan was requested at the presentation which excluded aortic dissection.

AVR surgery has been the treatment approach of choice until the more recent advent of TAVI which has revolutionized the treatment of AS patients [14]. Earlier studies that showed benefits with TAVI had clearly defined exclusion criteria which included anatomical pathologies such as unicuspidal or BAVs [2]. However, the use of TAVI has recently gone beyond these indications to include other clinical presentations too [14].

A meta-analysis among patients with BAV and no-BAV cohorts showed that there was no difference in 30-day mortality following TAVI (8.3% vs 9.0%; p=0.68) [15]. However, the unique morphological features of BAV, and the lack of consensus on the optimal sizing technique, pose a challenge when offering TAVI to such patients [14,15].

Another consideration in the index patient’s presentation is the fact that he was Jehovah’s witness, who stated that he would not want to have a blood transfusion peri- or postoperatively. The possibility of perioperative blood loss further influenced decision-making during the multidisciplinary team meeting. Patients’ autonomy is always paramount to treatment decisions. A similar scenario has been previously reported among this religious sect with complex AS [16].

Bleeding events after surgical AVR and transcatheter AVR are common and may lead to unfavorable outcomes. This risk was reported by Genereux et al. to be higher among patients who had surgical valve replacement compared to those who had TAVI [17]. Smaller valve size, longer sternal incisions, and longer procedural time were major determinants of outcome, while higher rates of migration or embolization of the prosthesis, and conversion to open-heart surgery, were associated with our TAVI outcome [17].

The index patient also had coexisting temporal lobe epilepsy and was on sodium valproate and primidone as antiepileptic medications. He was placed on aspirin after the TAVI procedure. The need for long-term anticoagulation with vitamin K antagonists (VKA) if surgical AVR was the treatment modality as compared with TAVI also influenced the treatment decision. Aspirin is not known to significantly interact with these antiepileptic medications compared with anticoagulants like warfarin or non-VKAs [18]. Meta-analyses of several randomized control trials have reported a considerable reduction in bleeding risk without a significant difference in mortality when a single antiplatelet (aspirin) is used post-TAVI, compared to dual antiplatelets [18,19]. It was reported from these studies that anticoagulants are only recommended if the patient had other indications for their use. Some studies are increasingly showing benefits with novel oral anticoagulants following surgical AVR [19]. However, most antiepileptic medications are known to interact with warfarin and non-VKAs, reducing their efficacy [18].

Finally, van Mourik et al. reported that the guideline-defined TAVI futility, which was defined as mortality within one year or no objective symptomatic improvement in New York Heart Association class, was present in 28.6% of patients following TAVI [20]. It is therefore recommended that a yearly follow-up until five years post-surgery be arranged for our index patient, with a view to monitoring treatment outcome as well as the clinical consequence of the residual mitral valve regurgitation.

## Conclusions

AS commonly coexists with other anatomical peculiarities that may make therapeutic decisions challenging. Decision-making on which intervention is best for the patient can be further affected by individual patient characteristics. Patients with severe symptomatic AS being considered for valve intervention should be evaluated by a multidisciplinary team, with either referral to or consultation with a primary or comprehensive valve center. A multidisciplinary team approach considering the treatment in the best interest of the patient, that is supported with evidence, should be the standard of care. TAVI is beneficial in well-selected patients with BAVs who have conditions that may preclude them from having aortic valve surgery. The best interest of the patients should be considered at every time while autonomy is maintained.
